# Offsetting Expression Profiles of Prognostic Markers in Prostate Tumor vs. Its Microenvironment

**DOI:** 10.3389/fonc.2019.00539

**Published:** 2019-06-26

**Authors:** Zhenyu Jia, Jianguo Zhu, Yangjia Zhuo, Ruidong Li, Han Qu, Shibo Wang, Meiyue Wang, Jianming Lu, John M. Chater, Renyuan Ma, Ze-zhen Liu, Zhiduan Cai, Yongding Wu, Funeng Jiang, Huichan He, Wei-De Zhong, Chin-Lee Wu

**Affiliations:** ^1^Department of Botany and Plant Sciences, University of California, Riverside, Riverside, CA, United States; ^2^Department of Urology, Guizhou Provincial People's Hospital, Guangzhou, China; ^3^Department of Urology, Guangdong Key Laboratory of Clinical Molecular Medicine and Diagnostics, Guangzhou First People's Hospital, School of Medicine, South China University of Technology, Guangzhou, China; ^4^Department of Mathematics, Bowdoin College, Brunswick, ME, United States; ^5^Department of Pathology and Urology, Massachusetts General Hospital and Harvard Medical School, Boston, MA, United States

**Keywords:** prostate cancer, microenvironment, biochemical recurrence, tissue microarray, prognosis, offsetting expression

## Abstract

Diagnosis of the presence of tumors and subsequent prognosis based on tumor microenvironment becomes more clinically practical because tumor-adjacent tissues are easy to collect and they are more genetically homogeneous. The purpose of this study was to identify new prognostic markers in prostate stroma that are near the tumor. We have demonstrated the prognostic features of FGFR1, FRS2, S6K1, LDHB, MYPT1, and P-LDHA in prostate tumors using tissue microarrays (TMAs) which consist of 241 patient samples from Massachusetts General Hospital (MGH). In this study, we investigated these six markers in the tumor microenvironment using an Aperio Imagescope system in the same TMAs. The joint prognostic power of markers was further evaluated and classified using a new algorithm named Weighted Dichotomizing. The classifier was verified via rigorous 10-fold cross validation. Statistical analysis of the protein expression indicated that in tumor-adjacent stroma FGFR1 and MYPT1 were significantly correlated with patient outcomes and LDHB showed the outcome-association tendency. More interestingly, these correlations were completely opposite regarding tumor tissue as previously reported. The results suggest that prognostic testing should utilize either tumor-enriched tissue or stroma with distinct signature profiles rather than using mixture of both tissue types. The new classifier based on stroma tissue has potential value in the clinical management of prostate cancer patients.

## Introduction

After decades of research, prostate cancer remains one of the leading worldwide concerns in male health (1, 2). Three major challenges need to be better addressed through biomarker studies to improve the management of the disease and save lives—early detection (3, 4), accurate prediction of patients' outcomes (5, 6), and development of effective personalized treatments for different types of prostate tumors (7). In the current study, we focused on the identification and verification of biomarkers that are associated with patients' outcomes, i.e., whether biochemical recurrence (BCR) will occur after prostatectomy, or how soon the disease will come back (e.g., time to BCR) if the disease does recur. Such biomarkers, once identified and validated, may be used in clinical applications to distinguish the patients who require surgery or/and adjuvant therapy from the patients who only need active surveillance (8–10).

Gene expression profiles have been widely scrutinized for years in order to develop expression signatures for the prediction of BCR status (3). Nevertheless, very few clinically applicable expression signatures have been developed, including Prolaris (46-gene test) from Myriad Genetics Inc., 22-gene test from Decipher Inc., and Genomic Prostate Score (17-gene test) from Oncotype DX, all of which still leave great room for improvement. The difficulty for development of prognostic markers from prostate tumor tissue could be due to the heterogenic nature of the prostate tumors and also because of the offsetting expression of signature genes in the tumor vs. its microenvironment, which makes it difficult to utilize these gene markers in a mixture of tissues (a new discovery of this study). A statistic-relevant explanation could be owing to the relatively small sample size in individual studies such that in each study the sample did not fully represent the heterogeneous population of prostate tumors (3, 6, 11–13). Another possibility could be that gene expression is controlled by many complicated biological systems, such as subtle gene networks (interaction among genes), epigenetic modification, small RNA interference, and transcriptional modification such as alternative splicing (14, 15). Multiple biochemical steps are involved from DNA to protein through the Central Dogma. It has been well-assumed that proteins (products of gene transcription) directly reflect genes' functions which play critical roles in biological processes *in vivo*. However, there is not always a correlation between protein expression and mRNA expression (16, 17). Therefore, the quantification of protein might be better correlated with the phenotypes of interest, such as aggressiveness of the cancer. In many studies, the expression of a protein has been used as a measure of a disease phenotype, for example, prostate-specific antigen (PSA), prostate-specific membrane antigen (PSMA), prostatic acid phosphatase (PAP) and prostate stem cell antigen (PSCA)/ alpha-fetoprotein (AFP), carcino-embryonic antigen (CEA), and cancer antigen (CA-125) (18–23). Therefore, predictive models based on the progression-associated antigens (protein markers) may potentially have increased prognostic power compared to the models that are solely based upon the gene expression profiles.

In our previous studies, we have demonstrated the prognostic potential for six proteins (FGFR1, FRS2, S6K1, LDHB, MYPT1, and P-LDHA) using a tissue microarray (TMA) system that was developed in-house at Massachusetts General Hospital (MGH) (5, 24–26). All the protein expression data were obtained through manual evaluation of the TMAs by pathologists, which may have led to possibly inconsistent readings and an artificially enlarged variation of the data (27). These six proteins showed differentially expressed levels between relapsed tumors and non-relapsed tumors. Independent RT-PCR experiments demonstrated similar expression patterns in mRNA levels for these proteins. In the current study, we explored the possibility of extending the application of these prognostic markers to the tumor microenvironment. It is difficult to diagnose the presence of prostate tumors, especially in their early stage, with biopsies due to their small volumes. Thus, methods have been developed to detect prostate tumors using surrounding tissues (with significant larger effective volumes) based on the assumption that tumor-adjacent tissues are restructured by the nearby tumor via paracrine (3, 28–30). We hypothesized that tumor microenvironments respond differentially to aggressive tumors *vs*. indolent tumors, and such differences, including differentially expressed proteins, may be used as signatures for prognosis.

To test this hypothesis, we reevaluated these MGH TMAs by only selecting the non-tumor regions that are adjacent to tumors. The array data of these six proteins were individually analyzed using an automated Aperio Imagescope system, as described in our previous study in PCa diagnosis with tumor-free cells (30), rather than manual evaluation. The Aperio system utilized a single standard to read the TMA image and calculated the average immunohistochemistry reaction intensity for each sample on the TMA, yielding reliable expression data. Additional within array normalization was carried out to remove potential systematic differences between various batches of array reactions. The objective data were then analyzed using various statistical methods to evaluate the association between each of the protein markers and BCR status or time to BCR. We found in tumor-adjacent stroma that FGFR1 was negatively associated with the risk of BCR (*p*-value = 0.005), MYPT1 was positively associated with the risk of BCR (*p*-value = 0.008), and LDHB showed a positive-association tendency (*p*-value = 0.164); however, such association patterns, either positive or negative, were completely opposite to what had been reported in tumor tissues (5, 24–32). We refer to “opposite” the distinct association patterns between the protein expression and risk of recurrence in the tumor site *versus* the tumor-adjacent stroma site. This interesting phenomenon may reflect the intricate interaction between tumor and microenvironment, the understanding of which may benefit clinical diagnosis and prognosis of the disease. Moreover, the results suggested prognosis of prostate cancer should be based on either highly enriched tumor tissue or stroma tissue (close to tumor) with distinct signature profiles rather than a mixture of both tissue types.

A newly developed approach, named Weighted Dichotomizing, was used to train a predictive classifier using these three prognostic markers. The results showed an accuracy of 71% in predicting the BCR status for the patients using the classifier. The model has been verified using 10-fold cross validation. The properties of the new predictive classifier, its clinical potential, and the potential for improvement have been discussed.

## Materials and Methods

### Prostate Tissues and Tissue Microarray (TMA) Assays

TMAs include formalin-fixed and paraffin-embedded (FFPE) specimens from 241 patients who were confirmed to have PCa and received radical prostatectomy at MGH from September 1993 to March 1995 (5, 24–27).

The study was approved by the human study ethics committees at Massachusetts General Hospital (Boston, MA) and the Ministry of Public Health of the People's Republic of China. All specimens in this study were anonymously handled according to ethical and legal standards.

The clinicopathological characteristics of all cases represented on TMAs are summarized in Table S1, and the data for the 105 cases (36 aggressive cases and 69 indolent cases) are summarized in Table 1. Clinicopathological data including pre-operation PSA, Gleason scores (GS, reassigned based on the current grading recommendation provided by the International Society of Urological Pathology), American Joint Committee on Cancer (AJCC) T stage, surgical margin status, time to biochemical recurrence (BCR) or PSA failure, time to metastasis, and overall survival time have been collected. The time to BCR was defined as the time interval between initial operation and first appearance of two consecutive rises of PSA. The time to metastasis was defined as the time interval between the initial operation and the detection of metastatic sites. The overall survival time was calculated from the date of surgery to the data of the last follow-up or death. None of the patients or subjects recruited for the study had chemotherapy or radiotherapy before the surgery. All tissues were reconfirmed by HE staining.

**Table 1:** Characteristics of 105 selected cases.

**Clinicopathological feature**	**Cases selected**
Number	105
**AGE FOR PATIENTS ONLY (YEAR)**	
Minimum	45
Maximum	77
Median	62
**PRE-OPERATION PSA (ng/ml)**	
≤4	15(14%)
>4	72(69%)
N/A	18(17%)
**GLEASON SCORE**	
≤6	44(42%)
7	42(40%)
≥8	19(18%)
**AJCC PATHOLOGIC T STAGE**	
2 (T2)	70(67%)
3 (T3)	35(33%)
**SURGICAL MARGIN STATUS**	
Negative	57(54%)
Positive	48(46%)
**METASTASIS**	
Negative	93(89%)
Positive	12(11%)
**OVERALL SURVIVAL**	
Alive	85(81%)
Die	20(19%)
**BIOCHEMICAL RECURRENCE**	
Negative	69(66%)
Positive	36(34%)

Immunohistochemical staining of formalin-fixed and paraffin-embedded sections was performed using a standard immunohistochemistry (IHC) protocol. Briefly, after deparaffinization and rehydration using a Leica autostainer XL ST5010 system, the TMA slides were pretreated with 10 mM sodium citrate buffer (pH 6.0) for 5–10 min in a microwave for antigen retrieval. The endogenous peroxidase was quenched by adding the hydrogen peroxide (3% H_2_O_2_ in 70% methanol) at room temperature for 15 min. After washing, the slides were blocked for 30 min. The blocking buffer was removed and the slides were then incubated for 1 h with primary antibodies (FGFR1, FRS2, LDHB, MYPT1, P-LDHA, and S6K1), respectively, with the optimized dilutions at room temperature. The catalog numbers for these six antibodies are ab10646 (Abcam Co Ltd, USA), sc-8318 (Santa Cruz, CA, USA), ab85319, (Abcam Co Ltd, USA), ab59235 (Abcam Co Ltd, USA), 8176 (Cell Signaling Technology, CA, USA), and ab32359 (Abcam Co Ltd, USA), respectively. The optimized dilutions for the six antibodies in the TMA reactions are 1:200, 1:200, 1:200, 1:300, 1:200, and 1:50 respectively.

Slides were washed with the 1×PBS solution and further incubated with DAKO Envision+/HRP for 30 min at room temperature. Detection was based on the use of the 3, 3′-diaminobenzidene as instructed (DAB kit, DAKO, Denmark). Slides were counterstained with hematoxylin before microscopic analysis. An H-Score was initially calculated based on scoring of stained cells according to published method (33).

### Image Analysis

The expressions of each protein in a TMA were measured by analyzing the staining signal intensity using Aperio image scope v11 (Aperio, USA). Briefly, in Aperio Imagescope windows, epithelial cancer cells and tumor-free micro-environmental area were compartmentalized by an experienced pathologist using pen tool, based on typical pathological features. The brown staining (positive) in the intensely stained image and the blue staining (negative) in the least intensely stained area were selected for further data processing. The subsequent staining intensity was measured as the densitometry of the digital image (×400), and the counted positive pixels were transformed to three intensity bins.

**Figure 1:**
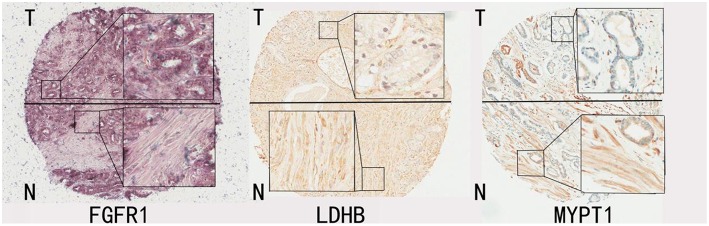
Representative IHC images for FGFR1, LDHB, MYPT1. The tissue regions labeled with T mainly represent tumor enriched area from patients' prostate glands; the tissue regions labeled with N represent tumor stroma areas of the prostate glands from the same patients.

A total of 181 tumor-bearing cases were considered in the study. For each case, tumor areas were first identified by an experienced pathologist with the aid of the Aperio Imagescope system. The pen tool in the Aperio Imagescope system was then used to select stroma that were close to tumor regions from each IHC image (shown in Figure 1), and the image data were then translated to numerical data, such as intensities of positive signal, intensities of negative signal, number of positive signals, and number of negative signals. The average intensity, which is the ratio of the sum of the intensities of positive signals (weak positive, positive, and strong positive) and the sum of the number of positive signals (weak positive, positive, and strong positive), is calculated and used for further statistical analysis.

### Redefinition of Study Cases

From the 181 tumor-bearing cases, we redefined 36 aggressive cases as the patients who had experienced BCR within 3 years after surgery removal of the gland and 69 indolent cases as the patients who did not show BCR for 6 or more years after the surgery.

### Basic Statistical Analyses

Pearson's Correlation Analysis was used to evaluate the relationship between the protein markers and the clinical variables. Survival analyses including Cox regression model and Kaplan-Meier were used to assess the association between protein markers and time to BCR. ROC curve and area under the curve (AUC) were used for the evaluation of the classification model. A *p*-value ≤ 0.05 was used for claiming a significant result for the statistical tests. Box plot and density plot were used for examining the distribution of the expression levels of each protein based on the patient's disease phenotype variables.

#### Weighted Dichotomizing Algorithm

We proposed to develop a composite predictive classifier using a multi-marker signature when different markers have various levels of predictability. The training set was first divided into a few subgroups based on a certain observed criterion, for example the binary clinical outcome of BCR status in the current study (aggressive group indicated by 1 and indolent group indicated by 0). For each marker, we sorted the patient cases based on the marker's expression level from lowest to highest. We sequentially used each of the sorted expression values of the marker as a cutoff to define predicted aggressive cases and predicted indolent cases. If the marker represents a protein product of an oncogene (i.e., higher expression levels are associated with more aggressive cases), the training cases with expression levels less than the cutoff were defined as predicted indolent cases and the training cases with expression levels greater than the cutoff were defined as predicted aggressive cases. If the marker is a product of a tumor suppressor gene, we defined the predicted indolent cases and predicted aggressive cases in the opposite way. The predicted indolent/aggressive classifications were then compared to the observed indolent/aggressive classifications to calculate the classification accuracy. Note that each cutoff was associated with a classification accuracy rate. In the process of developing a composite classifier, the cutoff with the highest classification accuracy was selected as the optimal cutoff for the marker, and the highest classification accuracy was used to calculate the weight for the marker in the composite classifier. A composite multi-marker classifier consisted of two components for classification calculation, i.e., the optimal cutoffs and the weights for the markers. Two steps were involved in the classification calculation when the composite classifier was applied to a test patient case. Suppose the composite classifier was composed of *k* markers or proteins (for example, *k* = 3 in the study because 3 proteins were analyzed). First, for the *i*th marker, we used the optimal cutoff to predict the patient outcome S_*i*_, with S_*i*_ = 1 for aggressive case and S_*i*_ = 0 for indolent case, where *i* = 1 to *k*. The weighed score for the outcome was calculated using the following formula:

(1) $$\begin{array}{r} S=\overset{k}{\underset{i=1}{\sum }}S_{i}w_{i}, \end{array}$$

where w_*i*_ was the weight for the *i*th marker which was calculated as:

(2) $$\begin{array}{r} w_{i}=\frac{\alpha_{i}}{\sum_{i=1}^{k}\alpha_{i}}, \end{array}$$

where a_*i*_ was the highest classification accuracy that was achieved for the *i*th marker in the training process. The test patient was predicted as an aggressive case if *S* ≥ 0.5 and predicted as indolent case if *S* < 0.5.

## Results

### Quantifying Expression Levels of Protein Markers Using an Aperio System

The images of selected tumor-adjacent stroma regions on all the tissue microarrays were analyzed and transformed to numerical expression values (average intensity *I*) using an Aperio System (see Figure 1). Normalization had been carried out for each sample within a TMA using the following formula

(3) $$\begin{array}{r} I^{\prime }=\frac{I-M}{R} \end{array}$$

where *I* and *I'* are the original and adjusted intensities, respectively, for a sample, *M* is the median intensity value for all the samples on the TMA, and *R* is the range of the intensity values for all the samples on the TMA. We only selected tumor-bearing tissues from a total of 181 patients who did not receive any adjuvant therapies after surgery for the study.

### Statistical Analysis of the Association Between Markers and Clinical Variables

**Figure 2:**
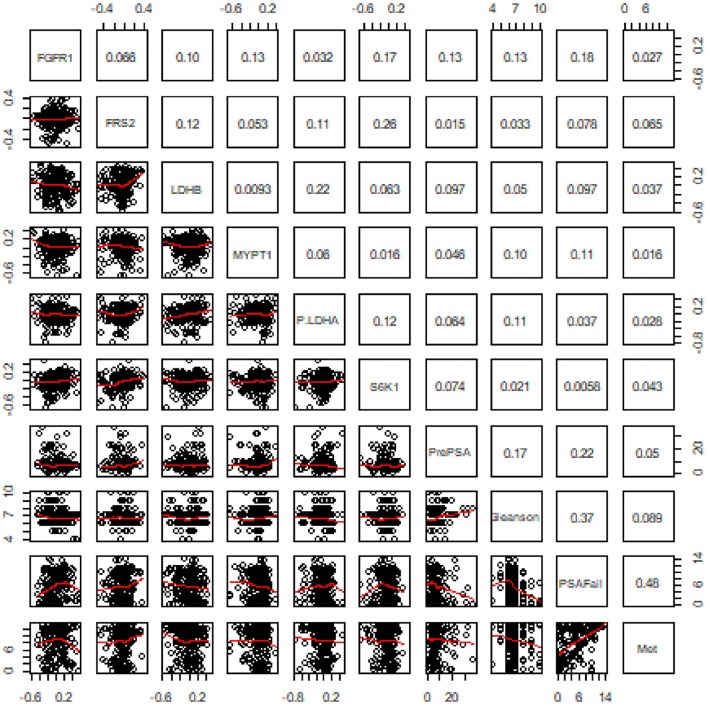
Results of pairwise correlation between six proteins and four clinical variables. The numbers in the grids are correlation coefficients in the pairwise correlation analysis.

Pairwise Pearson's correlation was first examined among the six proteins and with four clinical variables including pre-operation PSA, Gleason score, time to biochemical recurrence (BCR), and time to metastasis. No significant correlation was detected between any protein marker and the four important clinical variables which have been widely utilized in disease management (Figure 2).

**Figure 3:**
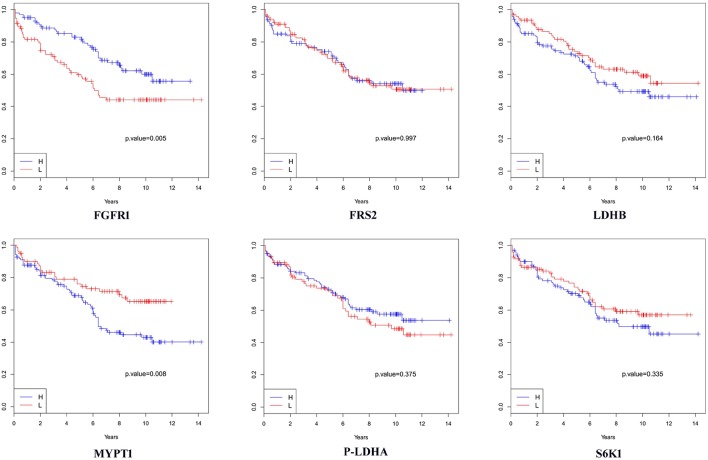
Survival analysis in terms of biochemical recurrence when tumor-adjacent stroma tissues were used. H: the subgroup of cases with expression levels greater than the median value. L: the subgroup of cases with expression levels less than the median value.

**Figure 4:**
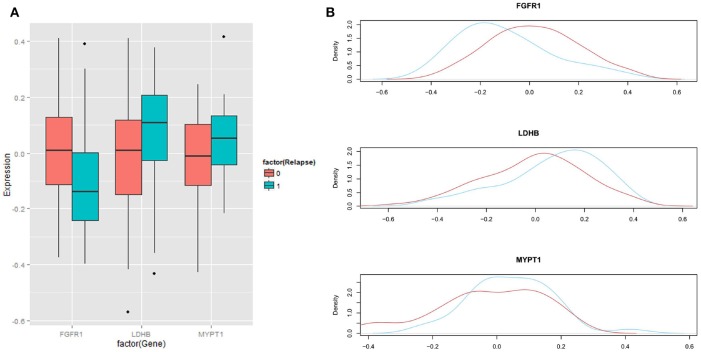
Distribution of expression levels in regard with BCR status. **(A)** Boxplots showing the differences in expression of three proteins between aggressive cases (labeled as 1 or blue) and indolent cases (labeled as 0 or red). **(B)** Distributions of expression of three proteins between aggressive cases (blue) and indolent cases (red).

We then analyzed the association between each of these 6 proteins and patients' biochemical recurrence (BCR) time using survival analysis. The patient cases were divided into two groups (H and L) based on the median expression value for the protein (5, 25, 34–41); the cases with expression levels greater than the median expression value were placed in group H and the cases with expression levels less than the median expression value were placed in group L. Kaplan-Meier curves were plotted in Figure 3 and *p*-values indicating the level of difference in survival between H and L groups were calculated using a Cox regression model (42). The results showed that FGFR1 (*p*-value = 0.005) and MYPT1 (*p*-value = 0.008) are significantly associated with the time to BCR, and LDHB (*p*-value = 0.164) is also relevant to the aggressiveness of the disease (Figure 3), which is consistent with the boxplots in Figure 4. However, the outcome-associations for these three proteins in tumor associated stroma are completely opposite to what we have observed in tumor tissues.

### Evaluation of Three Markers Jointly Using Weighted Dichotomizing Method

**Figure 5:**
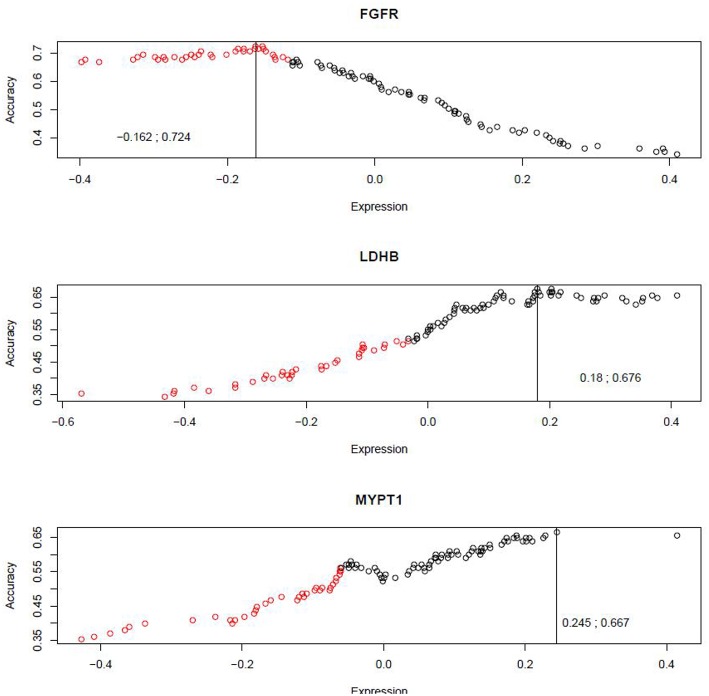
Systematic search for the optimal cutoffs. The x-axis represents the expression scores for protein markers, and the y-axis represents the classification accuracies achieved by various cutoff expression values.

In order to evaluate the prognostic potential for these three protein markers simultaneously, we developed a novel algorithm, named Weighted Dichotomizing (WD), for developing a multi-marker predictive classifier. There is a total of 36 aggressive cases and 69 indolent cases in the training set (see Materials and Methods). For each of the three proteins, we sorted the patient cases based on the expression level of the protein and then systematically searched for the threshold expression value for this protein that optimally distinguished the aggressive cases from the indolent cases, i.e., the cutoff expression value with the highest classification accuracy (Figure 5).

**Figure 6:**
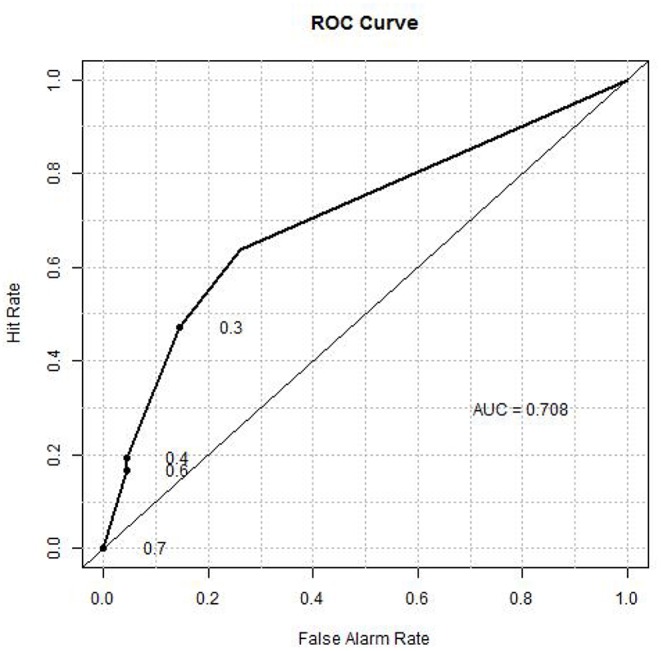
ROC curve showing the prognostic value of the 3-gene classifier.

A composite classifier was developed based on the calculation of the sum of the weighted classification using the three selected threshold values (as described in Materials and Methods). The weights used for the three markers were the highest classification accuracies (0.724, 0.676 and 0.667) that were achieved by the individual threshold expression values for the markers. When the classifier was applied to the training set, the classification accuracy or area under the curve (AUC) was 0.71 (see Figure 6 for ROC curve), with positive predictive value (PPV) and negative predictive value (NPV) being 0.70 and 0.69, respectively. We also verified the method using 10-fold cross validation as described below. The 105 patients (36 aggressive cases + 69 indolent cases) were arbitrarily divided into ten portions which were roughly of equal size with about 3 aggressive cases and 6 indolent cases in each portion. In each cross-validation step, we developed a classifier (as described above) using nine portions (~90%) of the cases and tested the classifier on the remaining one portion (~10%) which was not used for classifier development. We iteratively repeated this cross validation until each of the ten portions had been used exactly once for testing. Thus, we have predicted outcomes for each patient which can be compared with the observed outcomes to evaluate the performance of the classifier. The overall accuracy for the 10-fold cross validation was 0.66.

### Comparison of Prognostic Factors Using Survival Analysis

Univariate and multivariate analyses using the Cox Proportional-Hazards Model were performed to compare prognostic factors including the profiles the three 3 proteins (FGFR, LDHB, and MYPT1), the composite scores (WD Score) calculated from these protein profiles using the WD algorithm, and four primary clinical variables, i.e., Pre-OP PSA, Gleason score, Margin, and Stage (Table 2). The results of univariate analysis showed that the profiles of each of three proteins are significantly correlated with the time to BCR, however, the composite scores (WD Score) with the combined protein profiles demonstrated substantially stronger association with BCR, indicating an increase in prognostic power by combining prognostic biomarkers. The univariate analysis also showed that all four well-known pathological variables were individually associated with the time to BCR, with the Gleason score being the strongest predictor.

**Table 2:** Comparison of prognostic factors using cox proportional-hazards model.

	**Factor**	**Coef**.	**S.E. of Coef**.	**Z**	**P**
Univariate	FGFR	−3.0704	0.9774	−3.14	0.0017
	LDHB	1.659	0.874	1.9	0.058
	MYPT1	2.36	1.13	2.09	0.037
	*WD* Score	2.56	0.64	4	6.2*e*−05
	Pre OP PSA	0.0897	0.0225	3.99	6.5*e*−05
	Gleason	0.968	0.155	6.24	4.3*e*−10
	Margin	0.938	0.348	2.7	0.007
	Stage	1.083	0.334	3.24	0.0012
Multivariate	*WD* Score	2.4481	0.7145	3.43	0.00061
	Pre OP PSA	0.0532	0.0247	2.15	0.03123
	Gleason	1.0877	0.2174	5.00	5.6*e*−07
	Margin	−0.1812	0.4315	−0.42	0.67444
	Stage	0.0523	0.4625	0.11	0.90994

*Coef., estimated coefficient; S.E. of Coef., standard error of estimated coefficient; Z, z score; P, p-value*.

When the WD Score was combined with the four pathological variables in the multivariate analysis, Gleason, WD Score and pre-OP PSA were still statistically significantly associated with the time to BCR (in a descending order), whereas the previous associations of Margin or Stage with the time to BCR in the univariate analysis had vanished. These results indicated (1) the prognostic information provided by Margin or Stage in the univariate analysis may be well-represented by the data of either protein profiles or other clinical variables, and (2) combining protein profiles with nomograms based on clinical characteristics will potentially increase prognostic accuracy.

## Discussion

We investigated the profiles for six protein markers, FGFR1, FRS2, S6K1, LDHB, MYPT1, and P-LDHA, in tumor-adjacent stroma. The results indicated that FGFR1, MYPT1, and LDHB are relevant to disease progression, i.e., FGFR1 is negatively correlated with tumor progression whereas MYPT1 and LDHB are positively correlated with tumor progression in tumor-adjacent stroma. However, these correlations in tumor-adjacent stroma (in the current study) are completely opposite those in tumor tissue (as previously reported (5, 24–27, 31, 32)). Such a difference may reflect the intricate interaction between tumors and their associated stroma tissues. More importantly, the results suggested that prognosis of prostate cancer should be based on either highly enriched tumor tissue or stroma tissue (close to tumor) with distinct signature profiles rather than a mixture of both tissue types. This is because the expression of these marker genes in two tissue types may be offset in the mixture samples, yielding ambiguous test results.

Due to the heterogenic nature of the prostate tumors, it has been difficult to develop clinically useful biomarkers for prognosis. Compared to tumor tissues, tumor-adjacent stroma is much more genetically stable and homogenous. As the cancer develops, grows, and progresses, the stroma tissue in the surrounding microenvironment co-evolves into an activated state through continuous paracrine communication. Studies have suggested that tumors restructure surrounding stroma tissues such that these tumor-adjacent stroma tissues become quite different from the remote stroma, and such differences depend on the tumors' properties, i.e., aggressive tumors and indolent tumors (43, 44). However, research on prognostic markers in tumor-adjacent stroma has been inadequate. In this study, we mainly focused on the exploitation of prognostic markers in tumor-adjacent stroma using existing TMA data.

FGFR1 has been intensively studied in prostate cancer (45–47). In the normal human prostate gland, expression of FGFR1 is restricted to stroma and is not expressed in epithelial cells (48). Nevertheless, prostate tumors exhibit aberrant expression of FGFR1 in epithelial carcinoma cells (49, 50), and increased expression of FGFR1 seemed to be associated with aggressive tumors. However, contradictory results have been reported for the FGFR family—for example, down-regulation of FGFR2 is associated with neoplastic progression (49–52). Moreover, previous studies found that cloned FGF family epithelial cells from the non-malignant PCa, when implanted in rat hosts in the absence of stromal cells, can progress to malignant PCa (53). As a key regulator of vascular smooth muscle, MYPT1 is a member of the myosin phosphatase targeting protein (MYPT) family, which is most abundant in smooth muscle cells (54). Expression of MYPT1 is associated with many human diseases, including cancers (55–57). A previous study also revealed that MYPT1 could affect the cell cycle, migration, and adhesion processes of cancer cells (55). Our previous study indicated that MYPT1 stimulation could antagonize the pro-tumor effect induced by Mir 30d-upregulation in PCa epithelial cells (25). Nevertheless, our current study showed that MYPT1 in tumor stroma was positively correlated with tumor progression. As for LDHB, it is one of the subunits of LDH which catalyzes the reversible conversion of pyruvate to lactate. It has been reported that reduced expression of LDHB is associated with progression of PCa and other forms of cancer (58–60). A previous study showed that loss of LDHB expression in prostate cancer was due to promoter hypermethylation (32). However, our results indicated that LDHB was positively correlated with tumor progression in tumor-adjacent stroma, which was also opposite to the previous study in tumor tissues. As discussed above, studies are needed to disclose tumor-stroma interactions.

It has been routine that pathologists or specially trained personnel read the TMA image file to provide scores for evaluating the expression levels for the protein of interest. These scores often take ordinal form, for example, 0 for negative signal, 1 for weak signal, 2 for moderate signal, and 3 for strong signal. The ordinal scale does not provide precise measurement because the intermediate levels between the ordinal numbers are not represented. Moreover, different pathologists may use different standards which are based on individual experiences, sometime yielding discordant data. The Pearson's correlation test between the scores provided by three pathologists showed poor concordance between them (Figure S1). In the study, we evaluated the TMAs to provide quantitative data using an automated way through the image software Aperio image scope v11 (Aperio, USA), avoiding potential bias or erroneous scoring due to manual work. Additional normalization was used to remove systematical errors between different batches of the arrays to achieve reliable data. Thus, TMAs analyzed using different antibodies with different batches may be analyzed at the same time to increase analytic power and reduce manpower.

Simple Pearson's correlation could not identify any substantial association between protein markers or between any marker and clinical variables. The results indicated that the markers, if they are relevant to a certain clinical outcome, may not be linked to the clinical variable in a linearly related manner. For example, a threshold expression level may exist for a protein marker such that patients with protein expression higher than that threshold exhibit one phenotype whereas patients with protein expression lower than that threshold have another phenotype; however, no correlation can be detected if we simply calculate the Pearson's correlation coefficient based on all the patients. This was also true when we used the median expression level as a cutoff to subdivide the training set into two equal groups and checked the survival in regard to BCR. It is also in agreement with the various dominance-recessive relations between phenotypes that are due to the gene dosage, i.e., haplo-sufficient or haplo-insufficient (61). The survival analysis indicated that FGFR1 (tumor suppressor gene), LDHB (oncogene), and MYPT1 (oncogene) are all relevant to the time to BCR; however, the lack of correlation among these three markers themselves well-support the “threshold” theory. When we check the distribution of expression levels for these three proteins between BCR status (0 for non-relapse and 1 for relapse), there was a substantial overlap between the non-relapse group and the relapse group. The results suggested that to distinguish aggressive cases from indolent cases, we need to use both the BCR status (relapse/non-relapse) and the time to BCR to deal with the censored data. From a total of 181 tumor-bearing cases, we redefined 36 aggressive cases as the patients who had experienced BCR within 3 years after surgical removal of the gland, and redefined 69 indolent cases as the patients who did not show BCR for 6 or more years after the surgery. Indeed, the aggressive and indolent cases here refer to the early relapse cases and non-relapse cases with long follow-up period, respectively. The censored data in clinical studies can also be properly analyzed by the Cox Proportional-Hazards Model. Table 2 showed that the Cox regression can detect the associations between protein profiles or well-known pathological variables with the time to BCR which had been missed by Pearson's correlation analysis. The multivariate Cox regression analysis suggested that protein markers and clinical variables may correlate with disease outcomes in different manners; thus, combining different types of predictors has potential to increase prognostic power, which warrants future research.

We developed a new algorithm, named Weighted Dichotomizing, to uncover the association between the protein markers and the aggressiveness of the tumors that are embodied by the BCR status and the time to BCR. For each marker, we first identify the threshold (or cutoff value) that optimally separates aggressive cases from indolent cases with the highest classification accuracy. We then combined the markers to form a composite classifier by considering the different predictive potentials for the markers, i.e., the combined model put more weight on the protein markers that had achieved higher classification accuracies in training. The prognostic classifier trained using the proposed method achieved an overall accuracy of 71% based on only three protein markers, and the approach was verified by 10-fold cross validation, with an overall accuracy of 66%. Note that only 3 protein marks and 105 patient samples have been used for development of the model. The performance of the classifier will increase if more markers and more patient samples are used to develop the composite model.

The in-house TMAs by MGH may be used to identify and validate more potential protein markers which could be identified by mining the publicly available databases, such as The Cancer Genome Atlas (TCGA) (62). However, in order to translate to clinical use, other platforms for easily analyzing proteins are needed, for example Quantitative Infrared Westerns (63), reverse phase protein microarrays (64), or isobaric tags for relative and absolute quantitation (65). Multicolor staining technology should be applied to future study. In our own future study, we will identify and validate more protein markers using the MGH TMAs and develop a composite classifier with improved accuracy. Also, we will use the MGH TMAs to analyze a few proteins of house-keeping genes to outline the basal expressions for these reference proteins. Normalization based on these reference proteins, which are assumed to have stable expressions across disease statuses, will be used to refine the classifier algorithm. A fluorescent multiplex immunohistochemistry (mIHC) protocol may be developed to reduce the potential systematic bias (66–68). The well-established classifier algorithm can be used to calculate risk score based on the data that are generated from other lab-friendly platforms. TMAs with improved quality and with more patients being included will certainly help develop a classifier with improved performance in clinic.

The potential limitation of the study is that different scoring methods were used for tumor assay and stroma assay, i.e., tumor samples in the previous studies were scored manually by pathologists (5, 24–27, 34–36), whereas the stromal regions in the current study were digitally scored using the Aperio Imagescope system. Further validation is needed to employ the same scoring method (i.e., digital scoring) and assay to analyze both tumor tissues and stroma tissues in new patient cases. Compared to tumors, stroma appears to be more homogenous. There are multiple cell-types in stroma; however, predominant cells in the tumorous stroma are fibroblasts/myofibroblasts (69). In the current study, we used the pen tool in the Aperio Imagescope system to select tumor-adjacent stroma for the analysis. Advanced methods, such as microdissection, may be used to collect more homogeneous samples for verification.

## Conclusions

The results suggest that prognosis of prostate cancer should utilize either tumor-enriched tissue or stroma (close to tumor) tissue with distinct signature profiles rather than using a mixture of both tissue types. This is because the expression of these marker genes in the two tissue types may offset in the mixture samples, yielding ambiguous test results. More importantly, the new classifier based on stroma tissue has potential value in clinical management of prostate cancer patients.

### Statement of Translational Relevance

Our study indicated that the protein expression of three markers (FGFR1, LDHB, and MYPT1) were correlated with prostate cancer patients' outcomes (biochemical recurrence) in tumor-adjacent stroma, and such correlations were surprisingly opposite to those in tumor tissues. The results suggested prognosis of prostate cancer should be based on either highly enriched tumor tissue or stroma tissue (adjacent to tumor) with distinct signature profiles, rather than a mixture of both tissue types. This is because the expression of these marker genes in two tissue types may offset in the mixture samples, yielding ambiguous test results. We further evaluated the joint prognostic power of these three protein markers in tumor-adjacent stroma by using a composite classifier developed through a new algorithm, named Weighted Dichotomizing, which was verified by rigorous 10-fold cross validation. The new classifier demonstrated potential value in clinical management of prostate cancer patients.

## Ethics Statement

The study was approved by the human study ethics committees at Massachusetts General Hospital (Boston, MA) and the Ministry of Public Health of PR China. All specimens in this study were anonymously handled according to the ethical and legal standards.

## Author Contributions

W-DZ supervised the whole project and study and participated in study design and coordination. ZJ, JZ, and YZ analyzed data and wrote and revised the paper. RL, HQ, SW, MW, JC, and RM revised the figures and verified the results. ZL, ZC, JL, YW, FJ, HH, and C-LW collected the samples information and evaluated the score. All authors read and approved the final manuscript.

### Conflict of Interest Statement

The authors declare that the research was conducted in the absence of any commercial or financial relationships that could be construed as a potential conflict of interest.

## Supplementary Material

Table S1 Characteristics of all cases represented on the MGH TMAs.

Figure S1 Comparison of expression scores of three proteins manually evaluated by 3 pathologists.
