# Supplementary material for: Offsetting Expression Profiles of Prognostic Markers in Prostate Tumor vs. Its Microenvironment
Source: Front Oncol. 2019 Jun 26;9:539. doi: 10.3389/fonc.2019.00539 (PMC6611437; doi:10.3389/fonc.2019.00539)
Supplement: Table S1 — Characteristics of all cases represented on the MGH TMAs. [file Table_1.DOCX]

Table S1. Characteristics of all cases represented on the MGH TMAs.

| **Clinicopathological feature** | **Cases in TMAs** |
| --- | --- |
| **Number** | 241 |
| **Age for patients only (Year)** |  |
| Minimum | 45 |
| Maximum | 78 |
| Median | 62 |
| **Pre-operation PSA (ng/ml)** |  |
| ≤4 | 34（14%） |
| >4 | 162（67%） |
| N/A | 45（19%） |
| **Gleason score** |  |
| ≤6 | 100（41%） |
| 7 | 106（44%） |
| ≥8 | 35（15%） |
| **AJCC pathologic T stage** |  |
| 2 (T2) | 182（76%） |
| 3 (T3) | 59（24%） |
| **Surgical margin status** |  |
| Negative | 149（62%） |
| Positive | 92（38%） |
| **Metastasis** |  |
| Negative | 221（92%） |
| Positive | 20（8%） |
| **Overall survival** |  |
| Alive | 195（81%） |
| Die | 46（19%） |
| **Biochemical recurrence** |  |
| Negative | 153（63%） |
| Positive | 88（37%） |
