# Supplementary figures and images for: Offsetting Expression Profiles of Prognostic Markers in Prostate Tumor vs. Its Microenvironment
Source: Front Oncol. 2019 Jun 26;9:539. doi: 10.3389/fonc.2019.00539 (PMC6611437; doi:10.3389/fonc.2019.00539)

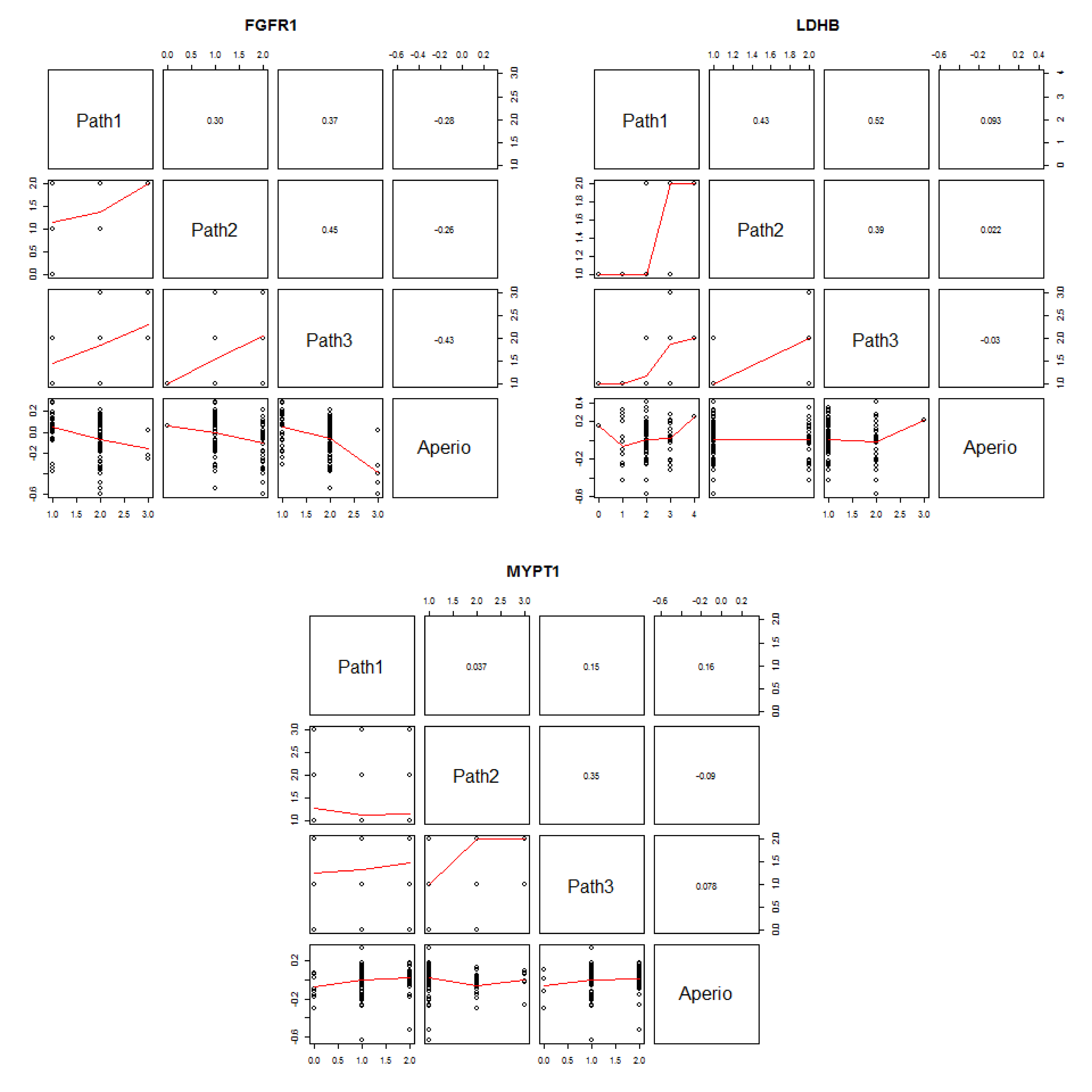

Supplement: Figure S1 — Comparison of expression scores of three proteins manually evaluated by 3 pathologists. [file Image_1.TIF]
